# Quality and Digestibility of Gluten‐Free Cookies Made From Rice Flour Substituted With Highly Enzyme‐Resistant Mung Bean Starch

**DOI:** 10.1155/ijfo/7689819

**Published:** 2025-12-12

**Authors:** Nguyen Thi Mai Huong, Nguyen Ngoc Thanh Tien, Nguyen Thi Lan Phi, Phan Ngoc Hoa, Pham Van Hung

**Affiliations:** ^1^ Institute of Biotechnology and Food Technology, Industrial University of Ho Chi Minh City, Ho Chi Minh City, Vietnam, hui.edu.vn; ^2^ Department of Food Technology, International University, VNU-HCM, Ho Chi Minh City, Vietnam, hcmiu.edu.vn; ^3^ Vietnam National University in Ho Chi Minh City, Ho Chi Minh City, Vietnam, hcmus.edu.vn; ^4^ Department of Food Technology, University of Technology, VNU-HCM, Ho Chi Minh City, Vietnam, ltu.se; ^5^ Department of Food Technology, Saigon Technology University, Ho Chi Minh City, Vietnam, stu.edu.vn

**Keywords:** digestibility, enzyme-resistant starch, gluten-free, mung bean, rice-based cookies

## Abstract

Gluten‐free cookie (GFC) made from rice flour is considered a healthy food for people suffering from celiac disease. This study was aimed at developing GFCs supplemented with highly enzyme‐resistant mung bean starch (HERS) and evaluating their physical properties, in vitro and in vivo digestibility, and organoleptic profiles. The HERS was used to substitute for rice flour at levels of 15%, 20%, 25%, 30%, 35%, 40%, and 45% in cookie making using stevia as a sugar substitute. The shape and surface of GFCs were identical to those of wheat‐based ones, but their color intensity gradually darkened with increasing amounts of HERS. The diameter of rice‐based cookies consistently decreased with a higher concentration of HERS added to the dough, whereas their thickness and volume initially increased but then decreased when the concentration of HERS exceeded 30%. However, their spread ratios exhibited an opposite trend to their thickness. Additionally, the hardness of GFCs increased with higher levels of HERS. These results revealed that rice‐based cookies substituted with 30% HERS exhibited the most appropriate color, appearance, spread ratio, and hardness among other cookies tested. This particular cookie was selected for further evaluation of digestibility and organoleptic profiles. The cookie contained a high amount of resistant starch (28.6%), a moderate concentration of slowly digestible starch (21.1%), and a low level of rapidly digestible starch (50.3%), resulting in a low glycemic index (51.7%). Furthermore, this product was highly rated, with an average score of 7 on a 9‐point scale for all sensory attributes. The rice‐based cookies substituted by 30% HERS were recognized as a gluten‐free and low‐GI product with excellent sensory attributes and health benefits.

## 1. Introduction

Gluten is crucial for baking as it strengthens the qualities of dough [1], but for some people, this protein stimulates autoimmune disorders in the small bowel, resulting in celiac disease [2]. Currently, approximately 1% of the global population suffers from the syndrome of celiac disease, with rising cases associated with advanced clinical examination [3]. Consequently, people with celiac disease must adhere to a lifelong gluten‐free diet [4]. Thus, the appreciable demand for gluten‐free foods necessitates that bakery producers create gluten‐free alternatives to satisfy this need.

Cookie, traditionally made from wheat flour, sugar, and fat, is a well‐known bakery product due to its palatability, long shelf life, and reasonable price, making it a preferred choice for nutrient distribution [5]. In cookies, fat and sugar primarily contribute to their crunchy texture, while gluten from wheat flour plays a minimal role [3, 5]. Therefore, there is growing interest in gluten‐free cookies (GFCs) made from cereals (rice, maize, sorghum, and millet), legumes, pseudocereals, and their blends for people suffering from celiac disease [3]. Punfujinda et al. [5] found that the purple sweet potato flour could be used to replace 30% corn flour to achieve the highest overall acceptability scores, indicating an optimal balance between nutritional enhancement and consumer preference for GFC. Rice flour, in combination with various other ingredients, has also been one of the most commonly used flours to develop GFCs [3]. In addition, pulse flour has also been widely used to mix with rice flour to enhance nutritional quality as pulses are rich in protein, fiber, resistant starch (RS), and healthy fats [6]. Yen et al. [6] reported that GFC made with rice flour substituted by 60% germinated mung bean flour showed similar baking loss, thickness, diameter, spread ratio, and lightness values to wheat flour cookies. However, the high starch content of rice flour can lead to a higher glycemic index (GI), posing risks for individuals with obesity or diabetes [7]. Therefore, current efforts focus on developing GFCs with a low GI using sugar substitutes or incorporating ingredients rich in RS.

RS, a fraction of starch that resists digestion by amylase, occurs naturally in whole grains (RS1) and native starches (RS2) or can be produced by modifying various starches through physical (RS_3_), chemical (RS_4_), or enzymatic processes. It offers health benefits comparable to dietary fiber, along with enhanced physicochemical properties for food processing [8]. A previous study confirmed that RS not only replaces traditional insoluble fibers but also provides unique benefits, making products less coarse and more palatable [9, 10], thereby enhancing consumer acceptance of RS‐enriched foods [11]. Additionally, many commercially available RS products of RS_3_ and RS_4_ are currently utilized as additives to improve food functionality or provide biochemical benefits [12, 13]. Among diverse sources, RS_3_‐containing mung bean starch, characterized by high amylose content and a C‐type crystalline category, demonstrates high retrogradation viscosity and stability with limited swelling properties. Wang et al. [14] affirmed that cookies with 25% heat–moisture‐treated mung bean starch exhibited comparable water retention and superior sensory qualities compared to those made with wheat fiber. In contrast, their study also showed that cookies supplemented with 5%–25% mung bean RS, produced by pullulanase debranching and retrogradation, contained higher levels of RS but demonstrated greater water retention, poor sensory evaluation, and less‐than‐ideal hardness values [14]. Therefore, the development of GFCs with a low GI, using rice flour and modified mung bean starch, should be further studied to achieve the right balance of ingredients and enhance overall product quality. In addition, replacing sucrose in cookie formulations with a natural, nontoxic sweetener such as stevia is also considered to lower the blood glucose level of the cookie product, making it suitable for individuals with diabetes and obesity [15]. This study is aimed at developing GFCs made from a mixture of rice flour and highly enzyme‐resistant mung bean starch (HERS), produced through enzymatic treatment combined with microwave irradiation, using stevia as a sugar substitute. The physical characteristics, textural properties, and appearance of the cookies with HERS substitution were examined, and the in vitro and in vivo digestibility and organoleptic profiles of a GFC substituted with an optimal HERS concentration were evaluated. Cookies made from wheat flour, using sucrose or stevia as a sweetener, were used as positive controls.

## 2. Materials and Methods

### 2.1. Materials

Mung bean (*Vigna radiata*), a hybrid variety named as DX044, was provided by the Legumes Research and Development Center, Field Crops Research Institute, Vietnam Academy of Agricultural Sciences. The ingredients used for cookie production consisted of wheat flour, Japonica rice flour, eggs, butter, salt, baking powder, vanilla powder, sucrose, and stevia, all of which were purchased from a local supermarket in Ho Chi Minh City, Vietnam. Pullulanase (1498 upun/g, Sigma‐Aldrich, St. Louis, Missouri, United States), *α*‐amylase (250 U/mL, Sigma‐Aldrich, St. Louis, Missouri, United States), and amyloglucosidase (3300 U/mL, Megazyme E‐AMGDF, Ireland) were used. Swiss white mice, aged around 5–7 weeks and weighing 25–28 g/each, were purchased from the Pasteur Institute in Ho Chi Minh City, Vietnam. Other chemicals were also purchased from the Sigma‐Aldrich Company (St. Louis, Missouri, United States).

### 2.2. Preparation of RS

Selected mung bean seeds were shiny, large, and uniform with a cylindrical shape. These crops had light yellow flesh, a P_1000_ of 60.1 g, a starch content of 43.1%, an amylose content of 32.9%, and an RS_1_ content of 13.0% [16]. Native mung bean starch was isolated from the seeds and then modified using a microwave‐assisted debranching treatment method, as previously published by Huong et al. [17]. The final product, labeled as HERS, had an RS_3_ content of 52.8% and an amylose content of 61.9% [17].

### 2.3. Baking Procedure

The list of ingredients and baking instructions for cookies followed the procedure of Deng (2019) with some modifications [18]. The recipes for making cookies were created by replacing rice flour with HERS at levels of 15%, 20%, 25%, 30%, 35%, 40%, and 45% as described in Table 1. Regarding the baking process, all flour and dry ingredients were first mixed well for 3 min. Next, butter was blended for 3 min, followed by the addition of eggs, the combination of flour and dry ingredients, and mixing for 5 min. The entire mixture was laminated to a thickness of 0.4 cm and then shaped using a circular cutter with a diameter of 5.5 cm. Baking was conducted at 170°C for the top and 180°C for the bottom for 17 min using an oven (MIWE Michael Wenz GmbH, Arnstein, Germany), after which the cookies were cooled at 22°C for 15 min. The obtained cookies were stored for further analysis.

**Table 1:** List of ingredients in cookie recipe.

	**Wheat flour (g)**	**Japonica rice flour (g)**	**HERS (g)**	**Sugar (g)**	**Stevia (g)**	**Eggs (g)**	**Butter (g)**	**Salt (g)**	**Baking powder (g)**	**Vani powder (g)**
WSC	1000	—	—	400	—	600	300	10	20	0.2
WC	1000	—	—	—	20	600	300	10	20	0.2
RC	—	1000	—	—	20	600	300	10	20	0.2
15RSC	—	850	150	—	20	600	300	10	20	0.2
20RSC	—	800	200	—	20	600	300	10	20	0.2
25RSC	—	750	250	—	20	600	300	10	20	0.2
30RSC	—	700	300	—	20	600	300	10	20	0.2
35RSC	—	650	350	—	20	600	300	10	20	0.2
40RSC	—	600	400	—	20	600	300	10	20	0.2
45RSC	—	550	450	—	20	600	300	10	20	0.2

Abbreviations: 15RSC, 20RSC, 25RSC, 30RSC, 35RSC, 40RSC, and 45RSC, cookies prepared from rice flour substituted with 15%, 20%, 25%, 30%, 35%, 40%, and 45% of HERS with stevia, respectively; HERS, highly enzyme‐resistant mung bean starch; RC, cookies prepared from 100% rice flour with stevia; WC, cookies prepared from 100% wheat flour with stevia; WSC, cookies prepared from 100% wheat flour with sugar.

In order to formulate the less sugar cookie, stevia was used as a sugar substitute. Cookies prepared from 100% wheat flour using sucrose were labeled as WSC, while cookies prepared from 100% wheat flour using stevia were noted as WC. These cookies were used as references. Rice cookies (RCs) were prepared from 100% rice flour using stevia, while cookies made from rice flour substituted with 15%, 25%, 30%, 35%, 40%, and 45% of HERS using stevia were named 15RSC, 20RSC, 25RSC, 30RSC, 35RSC, 40RSC, and 45RSC, respectively.

### 2.4. Cross‐Sectional Morphology of Cookies

The porosity structure of the cookies was examined through cross‐sectional morphology using a scanning electron microscope (JEOL JSM‐6480 LV, Jeol Ltd., Tokyo, Japan) at a magnification of ×100 [17]. A portion of the cookie was fractured into sizes of 1 × 1 × 0.5 cm. After lyophilization, the cookie surface was dyed with a layer of Au/Pd (60/40 *w*/*w*). The morphology of the cookie was viewed at an accelerating voltage of 10 kV.

### 2.5. Physical Properties of Cookies

The diameter, thickness, and spread ratio of the cookies were measured using Vernier electronic calipers (Mitutoyo, Kanagawa, Japan) according to the modified method of Sudha et al. [19]. Each cookie was evaluated at five different positions, and 10 cookies were randomly selected for each measurement. The spread ratio of the cookie was also computed by dividing the diameter by the thickness. Additionally, the volume of the cookies was determined using the rapeseed displacement method [20].

The hardness of the cookies was measured 24 h after baking using a CT3 4500 Food Structure Analyzer (Ametek Brookfield, Middleboro, Massachusetts, United States) with a TA07 Knife Edge probe (60 mmW). According to the research of Pareyt and Delcour [21], the hardness of the cookies was defined as the peak force recorded during compression at a distance of 40 mm. The test speed was set to 2.0 mm/s, with the sample sheared to 15 mm. The trigger force was 0.5 N, the holding time was 0 s, and the sample length was 15 mm.

The color attributes of the cookies were determined using a Minolta CR410 colorimeter (Konica Minolta Co., Tokyo, Japan) with reference to the standard illuminator D65 and a 10° viewing angle. The outputs were automatically recorded as *L*∗, *a*∗, and *b*∗ values, and the color difference (*Δ* *E*∗) was calculated using the equation described in previous research [22].

### 2.6. Proximate Compositions of Cookies

The proximate composition of the cookies was estimated using the standard approved procedures [23]. The sample (2.0 g) was dried at 105°C for 6 h in a forced‐draft oven (CE3F‐2, Shel Lab, Oregon, United States) according to the methods of AOAC 950.46. Protein content was determined using a Kjeldahl digestion system (KI 26, Gerhardt, Germany) based on the standard method of AOAC 917.09. Lipid content was determined by extraction with hexane for 6 h using a Soxhlet apparatus (AACC Approved Method 30–10). Ash content was determined by burning in a muffle furnace at 550°C for 3 h (AOAC 942.05). The total carbohydrate concentration was calculated by subtracting the concentrations of protein, lipid, and ash from the total dry matter as follows: total starch (*%*, db) = 100*%* − *%*protein (db) − *%*lipid (db) − *%*ash (db).

Total sugar content was determined based on a previously published method of Tian et al. [24] with slight modification. The dry cookie powder (200 mg) was mixed with 20 mL of 80% ethanol for 10 min at ambient temperature and then centrifuged at 5000 × *g* for 20 min. The extraction was done repeatedly three more times. After extraction, the supernatant was collected and evaporated at 50°C using a rotary evaporator (Eyela, N‐1300, Tokyo Rikakikai Co., Tokyo, Japan). Then, the residue was dissolved in 20‐mL distilled water with 0.5 g poly(vinylpolypyrrolidone) following the centrifugation at 5000 × *g* for 10 min. The supernatant was made up to a known volume and was assayed for total free sugars by the phenol–sulfuric acid method [24]. The energy of the cookies was computed from amounts of total sugar.

### 2.7. In Vitro and In Vivo Digestibility of Cookies

The in vitro starch digestibility of cookies was determined as previously reported by Hung et al. [20]. The cookie powder was digested using an enzyme solution of alpha‐amylase (1400 U/mL) and amyloglucosidase (13 AGU/mL). The total glucose (TG) concentrations of the hydrolysates after 20 and 120 min digestion were determined (G20 and G120, respectively) using the phenol–sulfuric acid method [24]. Then, the samples were intensively hydrolyzed with 7 M KOH and followed by digestion with amyloglucosidase (50 AGU/mL) and TG was evaluated. Rapidly digestible starch (%RDS), slowly digestible starch (%SDS), and resistant starch (%RS) of the cookie samples were calculated from the values of G20, G120, and TG as below equations:

RDS=G200.9×,SDS=G12020−G×0.9,RS=TG−G1200.9×.

The in vivo digestibility assay described by Hung et al. [20] was adapted to quantify the blood glucose response in mice after the ingestion of cookies. Fifteen 25‐g white mice (*Mus musculus domesticus*) of the Swiss line were individually housed in a light‐controlled (12‐h dark, 12‐h light) environment under fully nutritional conditions at the laboratory of the university based on the instructions of the Pasteur Institute in Ho Chi Minh City. After fasting for 24 h, the mice were fed 0.5 mL of a cookie suspension (7.5%, *w*/*v*) via oral administration using a specialized tool inserted directly into the stomach. Blood was collected from the tail of each mouse, and blood glucose levels were measured using a glucose test kit (Roche Ltd., Basel, Switzerland) before feeding and at 30, 60, 90, 120, and 180 min postfeeding. All steps in the assay were approved by the Institutional Animal Care and Use Committee of the International University, Vietnam National University in Ho Chi Minh City, Vietnam (#ACC201601). GI value was calculated based on the area under the blood glucose response curve, as previously reported by Hung et al. [7]. The incremental area under the curve was determined geometrically by summing the areas of the triangles and rectangles formed by the blood glucose response over time. The GI was then calculated as follows:

GI=area under the curve for starcharea under the curve for glucose.

### 2.8. Organoleptic Profiles of Cookies

The sensory evaluation of the cookies was conducted based on the approach of Giuberti et al. [25] with minor modifications. The organoleptic profiles of the cookies were scored by 100 untrained panelists aged 22–39 years, who consumed cookies at least twice per week. The study was reviewed and approved by the Scientific Committee of the Department of Food Technology, International University, VNU‐HCM (FT1#2024S2). Subjects were invited to participate in the sensory evaluation based on their interest in cookie consumption. Before participating in the study, informed consent was obtained from each subject. Panelists were seated in individual sensory booths at room temperature under white light and were given clear instructions regarding information and the parameters used for sensory evaluation before testing. Each participant was provided with three cookies made from a composite flour, and water was provided to cleanse the palate between sampling each cookie. The cookies were labeled with random three‐digit codes and served 1 day after baking. Panelists were asked to evaluate appearance (surface color, roughness, and presence of cracks), texture (initially chewiness, dryness, and stickiness), aroma, taste, and overall acceptability using a 9‐point favorability scale.

### 2.9. Statistical Analysis

The experiment was conducted using a completely randomized design (CRD) in at least triplicate. One‐factor analysis of variance (ANOVA) was performed on the obtained data to test and compare differences, with Tukey′s test at a significance level of *p* < 0.05 using IBM SPSS Statistics 22 (Statistical Product and Service Solutions, IBM, New York, United States) software.

## 3. Results and Discussion

### 3.1. Effects of HERS on Visual Color and Cross‐Sectional Morphology of GFCs

**Figure 1:**
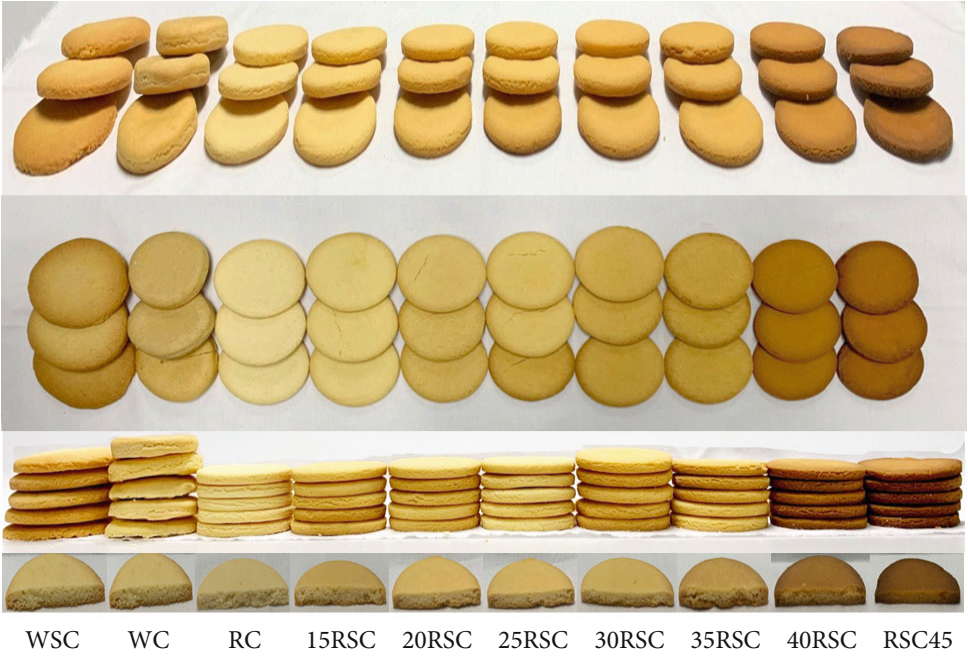
Photographs of gluten‐free cookies prepared from rice flour substituted with diverse concentrations of highly enzyme‐resistant mung bean starch. ^1^WSC, cookies prepared from 100% wheat flour with sugar; WC, cookies prepared from 100% wheat flour with stevia; RC, cookies prepared from 100% rice flour with stevia; 15RSC, 20RSC, 25RSC, 30RSC, 35RSC, 40RSC, and 45RSC, cookies prepared from rice flour substituted with 15%, 20%, 25%, 30%, 35%, 40%, and 45% of highly enzyme‐resistant mung bean starch with stevia, respectively.

**Figure 2:**
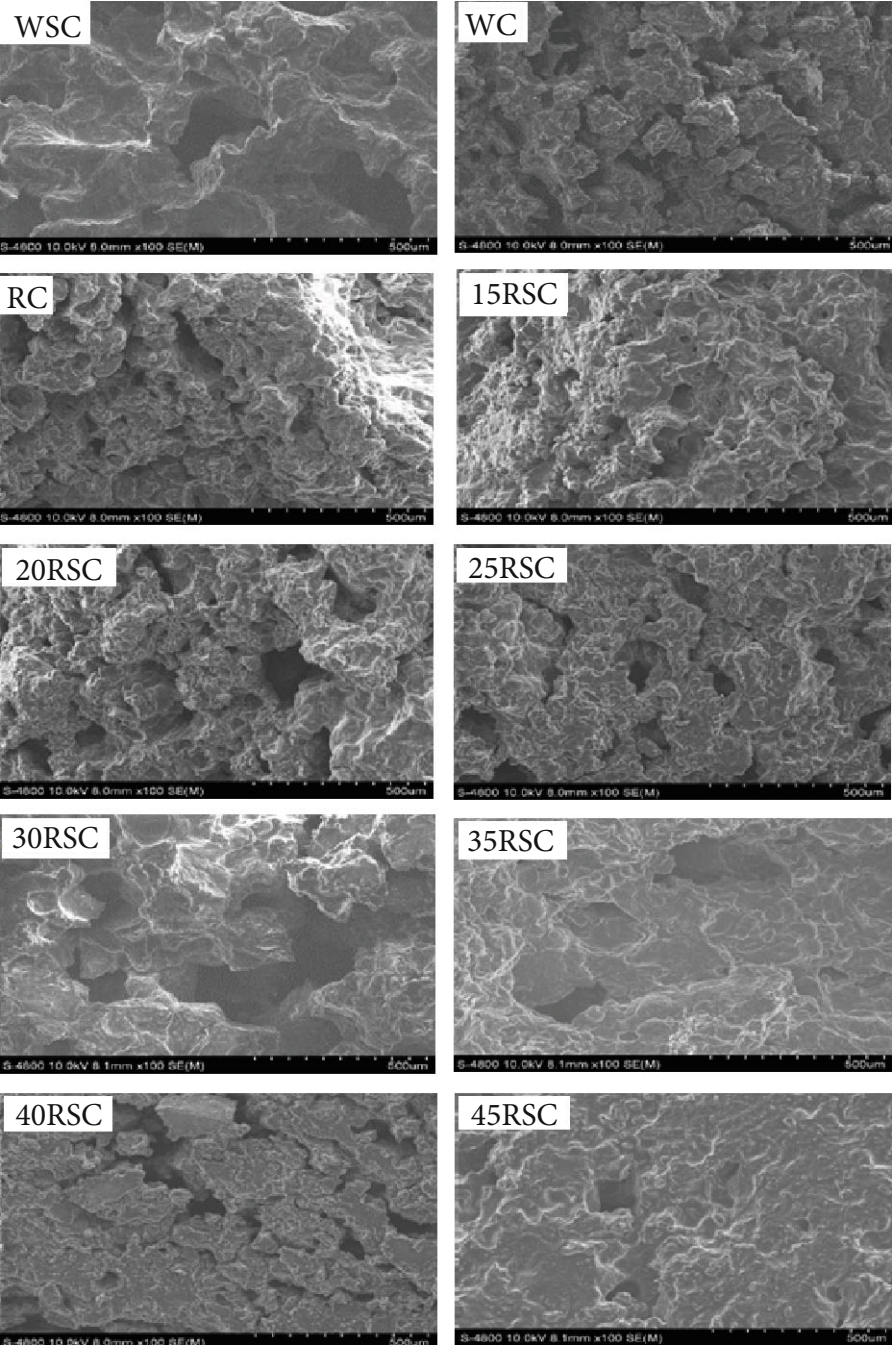
Cross‐sectional micrograph of gluten‐free cookies prepared from rice flour substituted with diverse concentrations of highly enzyme‐resistant mung bean starch. ^1^WSC, cookies prepared from 100% wheat flour with sugar; WC, cookies prepared from 100% wheat flour with stevia; RC, cookies prepared from 100% rice flour with stevia; 15RSC, 20RSC, 25RSC, 30RSC, 35RSC, 40RSC, and 45RSC, cookies prepared from rice flour substituted with 15%, 20%, 25%, 30%, 35%, 40%, and 45% of highly enzyme‐resistant mung bean starch with stevia, respectively.

The visual observation of cookies is one of the crucial benchmarks for evaluating consumers′ sensory perception of products, which is certainly impacted by the ingredients and technological process conditions [26, 27]. The images of GFCs are shown in Figure 1. Upon observation, the cookies made from wheat‐based or rice‐based flours using sucrose and stevia as sweeteners, along with a HERS substitution, exhibited diverse color, dimensions, and textures. Figure 2 indicates that the internal structure of these cookies visually reflects variations in porosity among the samples. The cookie made from wheat flour and sucrose (WSC) displayed a swollen gluten network, where the protein and starch granules were well mixed after gelatinization during baking. However, the cookie made from wheat flour and stevia (WC) as a sweetener had a more porous structure, with incompletely gelatinized starch granules and a disrupted gluten network. The cookie made from rice flour with HERS substitution had a rough structure and unswollen gluten network due to the low protein concentration and gluten quality. These changes have also been reported in previous studies [28, 29]. As a result, the cookies substituted with 30% HERS or 35% HERS (30RSC or 35RSC, respectively) exhibited a more continuous gluten network and gelatinized starch granules, similar to those of the WSC. In addition, the cookies substituted with higher levels of HERS (40% or 45%) had lower porosity and exhibited a more cohesive, denser internal structure. These results were consistent with the data on volume and hardness values presented in Table 2.

**Table 2:** Physical and mechanical properties of gluten‐free cookies prepared from rice flour substituted with diverse concentrations of highly enzyme‐resistant mung bean starch.

**Samples**	**Diameter (mm)**	**Thickness (mm)**	**Spread ratio**	**Volume (cm** ^**3**^ **)**	**Hardness (N)**
WSC	58.8 ± 0.4^g^	7.04 ± 0.08^g^	8.4 ± 0.1^b^	22.7 ± 0.4^g^	20.71 ± 0.56^d^
WC	50.6 ± 0.1^a^	9.62 ± 0.23^f^	5.3 ± 0.1^a^	20.1 ± 0.3^f^	25.17 ± 3.81^e^
RC	55.6 ± 0.1^f^	5.52 ± 0.13^c^	10.1 ± 0.3^e^	17.0 ± 0.5^c^	4.51 ± 0.40^a^
15RSC	54.6 ± 0.1^e^	6.15 ± 0.02^d^	8.9 ± 0.1^c^	18.1 ± 0.2^d^	6.86 ± 0.75^ab^
20RSC	54.8 ± 0.1^e^	6.23 ± 0.03^d^	8.8 ± 0.1^c^	18.5 ± 0.2^d^	10.61 ± 1.02^bc^
25RSC	55.4 ± 0.3^f^	6.32 ± 0.06^d^	8.8 ± 0.1^c^	19.1 ± 0.3^e^	12.30 ± 0.53^c^
30RSC	55.4 ± 0.1^f^	6.66 ± 0.06^e^	8.3 ± 0.1^b^	20.1 ± 0.3^f^	19.82 ± 2.10^d^
35RSC	53.5 ± 0.2^d^	5.58 ± 0.04^c^	9.6 ± 0.1^d^	18.0 ± 0.2^d^	20.98 ± 1.23^d^
40RSC	52.4 ± 0.2^c^	5.20 ± 0.04^b^	10.1 ± 0.1^e^	14.0 ± 0.3^b^	23.21 ± 1.76^de^
45RSC	51.7 ± 0.3^b^	4.77 ± 0.09^a^	10.8 ± 0.3^f^	12.0 ± 0.3^a^	25.34 ± 5.24^e^

*Note:* All data are the means of triplicate experiments ± standard deviations. Data followed by the various superscripts in the same column are significantly different (*p* < 0.05).

Abbreviations: 15RSC, 20RSC, 25RSC, 30RSC, 35RSC, 40RSC, and 45RSC, cookies prepared from rice flour substituted with 15%, 20%, 25%, 30%, 35%, 40%, and 45% of highly enzyme‐resistant mung bean starch with stevia, respectively; RC, cookies prepared from 100% rice flour with stevia; WC, cookies prepared from 100% wheat flour with stevia; WSC, cookies prepared from 100% wheat flour with sugar.

### 3.2. Effects of Substitution of HERS on Physical Characteristics of GFCs

The diameter and thickness of the cookies, measured after baking using the same prebaking mold, are shown in Table 2. The WC had a smaller diameter (50.6 mm) and greater thickness (9.62 mm) than the WSC (58.8 and 7.04 mm, respectively). These results imply the crucial impact of sugar interacting with gluten, leading to a continuous and strong gluten network [30], which directly affects the diameter and thickness of the cookies. The cookie made from rice flour and stevia (RC) had a smaller diameter (55.6 mm) and thickness (5.52 mm) than the WSC, whereas the cookies with substitutions of 15%, 20%, 25%, or 30% HERS (15RSC, 20RSC, 25RSC, and 30RSC) had unchanged diameters but greater thickness than the cookie made from rice flour and stevia (RC). Furthermore, the cookies substituted with more than 35% HERS showed a significant reduction in both diameter and thickness as the concentration of HERS increased. These results confirm the role of gluten in forming the structure and porosity of cookies [31]. The difference in the size of the cookies can be explained by an inverse correlation between cookie diameter and protein content [32], which suggests that lower protein concentrations lead to a larger diameter.

The spread ratio, or the ratio of diameter to thickness, is a key indicator of the degree of expansion and deformation of cookies [33]. Previous research revealed that the dough′s ability to rise and form cookies is closely related to a lower spread ratio [34]. Although the WC had a lower spread ratio than the WSC, the cookie made from rice flour and stevia (RC), with or without substitution of HERS (15%–45%), exhibited a significantly higher spread ratio than the WC. The increased spread ratio of the RC was attributed to its lower gluten content, higher dough water content, and greater water absorption by starch, which resulted in a more spreadable dough and reduced thickness. As the substitution concentration of HERS increased from 15% to 30%, the spread ratios of the cookies gradually decreased because their thickness consistently increased. However, when the concentration of HERS substitution exceeded 30%, the spread ratio of these cookies (35RSC, 40RSC, and 45RSC) showed the opposite trend to their thickness, due to a reduction in both diameter and thickness. The higher spread ratio of the cookies with HERS compared to the WSC can be explained by the poor swelling properties of RS compared to regular starch [27]. As a result, the spread ratio of 30RSC was not significantly different from that of the WSC. This result suggests that HERS can replace gluten in wheat flour while mitigating the disadvantages of not using sugar.

The WSC had a higher volume but lower hardness than the WC (Table 2). The volume of cookies made from rice flour and stevia (RC) was significantly lower than that of the WSC and WC. However, the volume of the cookies increased as the substitution concentration of HERS rose from 15% to 30%. When the substitution of HERS exceeded 30%, the volume of the cookies significantly decreased.

The hardness value, based on the breaking force, of the WC was significantly higher than that of the WSC, which is consistent with the results of the cross‐sectional view shown in Figure 2. Zoulias et al. [35] also reported that sucrose‐containing cookies had lower hardness than cookies made with other sweeteners. The lower hardness value indicates easier breakage of cookies, making them more fragile. The cookie made from rice flour had lower hardness than that made from wheat flour due to its lower protein content and gluten quality. However, as the substituted levels of HERS increased, the hardness values of the cookies also increased. The increase in the hardness value of cookies containing HERS can be explained by the poor moisture retention of RS, which leads to tightly packed molecules with stronger bonds [22]. Additionally, the lower water‐binding capacity and swelling force of RS contributed to better processing, resulting in harder and crisper cookies [36]. Among all GFCs, the hardness values of 30RSC and 35RSC were 19.82 and 20.98 N, respectively, which were not significantly different from that of the WSC. Thus, the most appropriate percentage of HERS added to dough to make cookies was 30%, as it is acceptable in terms of diameter, thickness, spread ratio, and desired hardness value among all GFCs. These outcomes align with previous studies on replacing 30% of buckwheat malt or green banana flour with rice flour in the production of GFC [31, 37].

### 3.3. Effects of Substitution of HERS on Color Profiles of GFCs

The color attributes of the GFCs, made from rice‐based flour using stevia as a sweetener and a HERS substitution, are presented in Table 3. The shape and surface of GFCs were identical to those of the WSC, but their color gradually changed. Among the wheat‐based and rice‐based cookies, the cookie made from rice flour and stevia (RC) had a brighter color, with the highest *L*∗ value, compared to the WSC and WC. This result can be explained by the difference in protein and sugar contents in the recipe [27]. Meanwhile, the *L*∗ value of GFCs decreased as the substituted levels of HERS increased, reflected by the darker color of the cookies. These findings are consistent with previous research, which revealed that the replacement of 10%–20% of gelatinized, retrograded, and extruded starch in the recipe of rice‐based cookies also resulted in a lighter color compared to the high starch‐substituted products [27]. Similarly, the *a*∗ and *b*∗ values of all samples showed positive values, tending toward red and yellow hues. Additionally, the greater the color difference (*Δ* *E*∗) compared to the WSC, the more noticeable the color difference in the cookies. The tendency for a darker color in cookies supplemented with a high concentration of HERS was consistent with previous studies on cookies substituted with modified maize flour using pressure cooking and enzymes [36] or annealed white sorghum starch [38]. The increase in color intensity of cookies could be attributed to the greater amounts of short reducing chains present in the HERS, which are activated by heat from microwave irradiation and enzymatic hydrolysis during processing. Previous studies confirmed that the presence of more reducing sugars caused a faster and darker color change in the products due to the Maillard reaction during baking [26, 36]. Based on the aforementioned results, the 30RSC exhibited the most appropriate color, appearance, spread ratio, and textural properties among the GFCs substituted with HERS. Thus, the 30RSC was selected for commercial production and continued study on its nutritional composition, starch digestibility, and organoleptic profiles, compared with the WSC, WC, and RC.

**Table 3:** Color analysis of gluten‐free cookies prepared from rice flour substituted with diverse concentrations of highly enzyme‐resistant mung bean starch.

**Samples**	**L**∗	**a**∗	**b**∗	**Δ** **E**∗
WSC	72.2 ± 0.4^e^	7.55 ± 0.57^e^	43.7 ± 0.7^h^	0.82 ± 0.16^a^
WC	70.4 ± 1.1^h^	6.02 ± 0.44^cd^	36.6 ± 0.5^c^	8.34 ± 0.12^b^
RC	82.6 ± 0.3^i^	0.20 ± 0.15^a^	31.3 ± 0.6^a^	15.25 ± 0.19^d^
15RSC	80.1 ± 1.4^g^	2.32 ± 0.47^b^	38.4 ± 0.8^d^	11.90 ± 1.35^c^
20RSC	74.8 ± 0.8^e^	6.30 ± 0.17^d^	39.8 ± 0.5^e^	7.10 ± 0.93^b^
25RSC	75.6 ± 0.4^e^	5.57 ± 0.22^c^	40.9 ± 0.3^ef^	8.34 ± 0.50^b^
30RSC	71.6 ± 1.3^de^	8.84 ± 0.20^f^	41.9 ± 0.3^g^	7.38 ± 0.45^b^
35RSC	69.8 ± 1.1^c^	10.15 ± 0.70^g^	42.3 ± 0.5^g^	7.96 ± 0.47^b^
40RSC	53.7 ± 1.0^b^	16.29 ± 0.38^h^	38.6 ± 1.1^d^	18.80 ± 0.68^e^
45RSC	48.5 ± 1.3^a^	16.37 ± 0.43^h^	34.3 ± 1.0^b^	23.21 ± 1.35^f^

*Note:* All data are the means of triplicate experiments ± standard deviations. Data followed by the various superscripts in the same column are significantly different (*p* < 0.05).

Abbreviations: 15RSC, 20RSC, 25RSC, 30RSC, 35RSC, 40RSC, and 45RSC, cookies prepared from rice flour substituted with 15%, 20%, 25%, 30%, 35%, 40%, and 45% of highly enzyme‐resistant mung bean starch with stevia, respectively; RC, cookies prepared from 100% rice flour with stevia; WC, cookies prepared from 100% wheat flour with stevia; WSC, cookies prepared from 100% wheat flour with sugar.

### 3.4. Proximate Analysis of the Cookies

The proximate compositions and energy provided by the cookies, made from wheat‐based and rice‐based flours with 30% HERS substitution, are illustrated in Table 4. The moisture content of all wheat‐based and rice‐based cookies after baking (1.32%–3.69%) was lower than 5%, which is suitable for preservation [33]. These results were consistent with previous studies on cookies containing abundant RS content, which reported that the moisture content of cookies with RS substitution ranged from 4.2% to 4.3% [22, 38]. Protein content varied among cookies due to the presence or absence of sucrose and the substitution level of HERS in the recipe. Although the protein content of the rice‐based cookie substituted with 30% HERS (30RSC) was lower than that of the WSC, the carbohydrate content of the 30RSC was also significantly lower than that of the other cookies, reflecting that the substitution of 30% HERS increased the amount of RS content in the formulation. The lipid and ash contents remained mostly unchanged. The total sugar content of the WSC was 17.2%, whereas this compound was not detected in the WC, RC, and 30RSC, the cookies using stevia as a sweetener. Variation in proximate compositions resulted in diverse energy levels. As a result, the 30RSC provided the lowest energy level (around 391 calories) compared to the other cookies.

**Table 4:** Proximate analysis of wheat‐based and rice‐based cookies.

**Samples**	**WSC**	**WC**	**RC**	**30RSC**
Moisture content (%)	1.32 ± 0.08	3.69 ± 0.04	2.45 ± 0.03	2.53 ± 0.06
Protein content (%)	7.23 ± 0.07	11.11 ± 0.09	9.53 ± 0.08	6.45 ± 0.18
Carbohydrate content (%)	69.3 ± 0.6	52.8 ± 0.8	60.4 ± 1.7	47.9 ± 1.6
Lipid content (%)	20.4 ± 0.85	19.8 ± 1.30	22.2 ± 0.97	19.3 ± 0.80
Ash content (%)	1.75 ± 0.06	2.63 ± 0.09	2.62 ± 0.11	2.65 ± 0.13
Total sugar content (%)	17.20 ± 0.7	Nd	Nd	Nd
Energy (Calories)	490	434	478	391

*Note:* All data are the means of triplicate experiments ± standard deviations. Data was calculated by the percentage of sample weight.

Abbreviations: 30RSC, cookies prepared from rice flour substituted with 30% of highly enzyme‐resistant mung bean starch with stevia; nd, not detected; RC, cookies prepared from 100% rice flour with stevia; WC, cookies prepared from 100% wheat flour with stevia; WSC, cookies prepared from 100% wheat flour with sugar.

### 3.5. In Vitro Digestibility of the Cookies

Table 5 illustrates the in vitro digestibility of wheat‐based and rice‐based cookies, expressed as the percentage of RDS, SDS, and RS. The cookies made from wheat flour using sucrose or stevia (WSC and WC, respectively) had higher SDS and RS contents but lower RDS content than those made from rice flour and stevia (RC). The cookie substituted with 30% HERS (30RSC) exhibited significantly lower concentrations of RDS (50.3%) but significantly higher concentrations of both SDS (21.1%) and RS (28.6%). The results indicate that the substitution of HERS significantly increased the SDS and RS content while decreasing the concentration of RDS. These outcomes were consistent with previous studies on cookies substituted with sweet potato starch treated with a combination of acid and heat‐moisture treatment [20], maize flour treated with a combination of enzyme and pressure [36], banana starch modified with citric acid [39], or hydrothermally treated waxy rice starch [40]. As a result, the 30RSC was found to have higher RS content than cookies substituted with 45% annealed white sorghum starch (12.3%) [38] or 60% commercial RS (11.23%) [22]. The increase in RS content of the GFC can be attributed to the high RS content of the HERS and its stability during the baking process [41]. As a result, the 30RSC was considered a beneficial GFC in terms of in vitro digestibility because the consumption of food containing at least 14% RS of the total starch is highly encouraged to provide health benefits [42, 43].

**Table 5:** In vitro digestibility of wheat‐based and rice‐based cookies.

**Samples**	**RDS (%)**	**SDS (%)**	**RS (%)**
WSC	75.1 ± 2.1^b^	16.8 ± 1.8^b^	8.1 ± 0.4^b^
WC	74.8 ± 1.1^b^	17.2 ± 1.2^b^	8.0 ± 1.4^ab^
RC	79.8 ± 1.8^c^	13.9 ± 1.1^a^	6.3 ± 1.2^a^
30RSC	50.3 ± 1.7^a^	21.1 ± 1.8^c^	28.6 ± 0.4^c^

*Note:* All data are the means of triplicate experiments ± standard deviations. Data followed by the various superscripts in the same column is significantly different (*p* < 0.05).

Abbreviations: 30RSC, cookies prepared from rice flour substituted with 30% of highly enzyme‐resistant mung bean starch with stevia; RC, cookies prepared from 100% rice flour with stevia; RDS, rapidly digestible starch; RS, resistant starch; SDS, slowly digestible starch; WC, cookies prepared from 100% wheat flour with stevia; WSC, cookies prepared from 100% wheat flour with sugar.

### 3.6. In Vivo Digestibility of the Cookies

**Figure 3:**
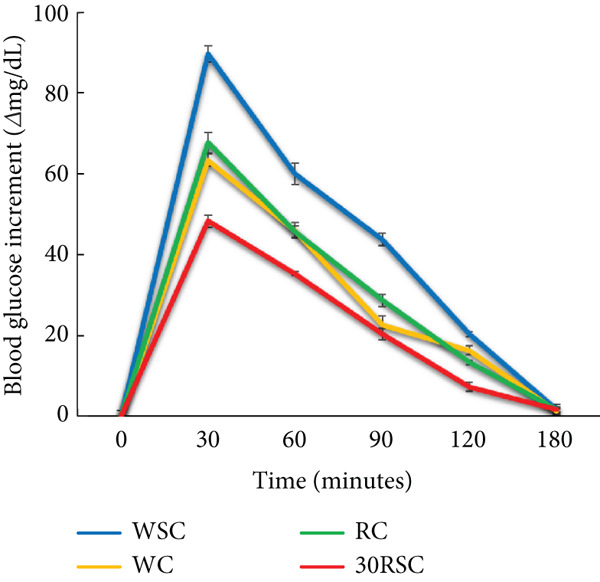
Blood glucose increment of cookies prepared from wheat and rice flour with and without substitution of highly enzyme‐resistant mung bean starch. WSC, cookies prepared from 100% wheat flour using sucrose as a sweetener; WC, cookies prepared from 100% wheat flour using stevia as a sweetener; RC, cookies prepared from 100% rice flour using stevia as a sweetener; 30RSC, cookies prepared from rice flour substituted with 30% of highly enzyme‐resistant mung bean starch using stevia as a sweetener. All data are the means of triplicate experiments ± standard deviations.

**Figure 4:**
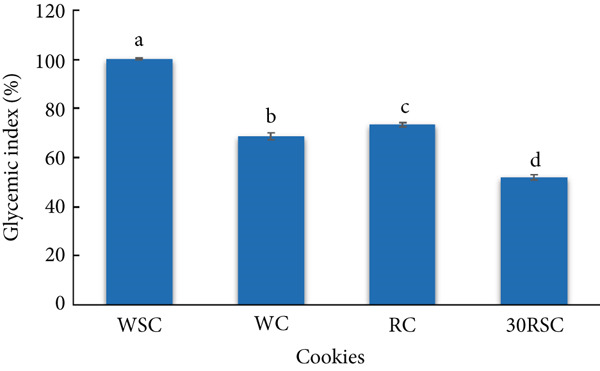
Glycemic index of gluten‐free cookies prepared from rice flour substituted with highly enzyme‐resistant mung bean starch. WSC, cookies prepared from 100% wheat flour with sugar; WC, cookies prepared from 100% wheat flour with stevia; RC, cookies prepared from 100% rice flour using stevia; 30RSC, cookies prepared from rice flour substituted with 30% of highly enzyme‐resistant mung bean starch with stevia. All data are the means of triplicate experiments ± standard deviations.

The in vivo digestibility of the cookies was expressed as the difference in blood glucose levels before and after feeding mice over digestion time (Figure 3) and their GI (Figure 4). All wheat‐based and rice‐based cookies exhibited a similar pattern of in vivo digestibility, where blood glucose response tended to increase substantially in the first 30 min, peaked at the 30th minute of digestion time, and then decreased considerably over the next 150 min. This trend was consistent with previous studies on cookies substituted with sweet potato starch treated with a combination of acid and heat–moisture treatment [20] or annealed white sorghum starch [38]. The results indicate that the WSC released the highest glucose level into the blood compared to the other cookies. In addition, the blood glucose level after consuming the RC was higher than that after consuming the WC. Consumption of the cookie with 30% HERS substitution (30RSC) resulted in a significantly lower blood glucose response compared to the WSC, WC, or RC throughout the digestion period. At the 30‐min peak, the blood glucose response in mice after consuming the 30RSC was 48.3 mg/dL, nearly half of that observed after consuming the WSC (89.7 mg/dL). Furthermore, the blood glucose levels in mice fed the 30RSC declined more slowly and steadily throughout the digestion period, from 31 to 180 min. The lower blood glucose response in mice after consuming the 30RSC may be due to its lower RDS and higher RS content compared to the other cookies.

The GI of the WSC, used as a reference, was 100% (Figure 4). The cookies that used stevia as a sweetener had a significantly lower GI than those that used sucrose. The GI of the 30RSC was 51.7%, significantly lower than that of the WSC, WC, and RC. Thus, the substitution of HERS remarkably reduced the GI of the final products. The lower GI of the 30RSC can also be explained by its lower glucose response level compared to the other cookies. Previous studies have revealed that foods with a high GI (> 70%) are associated with Type II diabetes, cardiovascular diseases, and obesity, while products with medium GI (55%–69%) and low GI (< 55%) have been reported to help prevent metabolic disorders, such as reducing blood protein levels, oxidative stress, and preventing colorectal cancer [39, 44]. Thus, the WSC, WC, and RC are considered high‐GI foods, whereas the 30RSC is considered a low‐GI food.

### 3.7. Organoleptic Profiles of the Cookies

Table 6 presents the organoleptic profiles of wheat‐based and rice‐based cookies, with and without HERS substitution, including appearance, texture, flavor, taste, and overall acceptability. Both WSC and 30RSC were rated similarly, with average scores exceeding 7 for all sensory attributes, indicating a high level of likability for these cookies. In contrast, the sensory scores for WC and RC were substantially lower than those of WSC and 30RSC. For the appearance attribute, WC scored less than 4, reflecting a low preference likely due to the deformation and shrinkage observed in wheat‐based cookies with sugar substitution. Differences in the texture scores may be attributed to variations in the volume and hardness of the cookies. Regarding overall acceptability, 30RSC received a slightly lower score than WSC but was still rated favorably, with an average score of 7 on a 9‐point scale. These findings align with previous studies on cookies substituted with corn starch [45]. Overall, the current evaluation suggests that HERS can effectively substitute up to 30% of rice flour to produce cookies with desirable sensory attributes and strong overall acceptance.

**Table 6:** Sensory evaluation of wheat‐based and rice‐based cookies.

**Samples**	**Appearance**	**Texture**	**Flavor**	**Taste**	**Overall acceptability**
WSC	7.72 ± 1.13^c^	7.57 ± 1.15^c^	7.45 ± 1.21^c^	7.44 ± 1.13^b^	7.42 ± 1.07^d^
WC	3.87 ± 0.80^a^	5.50 ± 1.19^a^	5.91 ± 1.15^a^	6.19 ± 1.56^a^	4.36 ± 1.03^a^
RC	6.39 ± 1.06^b^	5.91 ± 1.28^b^	6.81 ± 1.45^b^	6.16 ± 1.37^a^	5.78 ± 1.12^b^
30RSC	7.51 ± 1.30^c^	7.32 ± 1.17^c^	7.09 ± 1.25^b^	6.48 ± 1.34^a^	7.02 ± 1.07^c^

*Note:* All data are the means of 100 panelists ± standard deviations. Data followed by the various superscripts in the same column are significantly different (*p* < 0.05).

Abbreviations: 30RSC, cookies prepared from rice flour substituted with 30% of highly enzyme‐resistant mung bean starch with stevia; RC, cookies prepared from 100% rice flour with stevia; WC, cookies prepared from 100% wheat flour with stevia; WSC, cookies prepared from 100% wheat flour using sugar.

## 4. Conclusion

The results indicate that GFC was successfully developed using rice flour supplemented with various concentrations of HERS. Among all the rice‐based cookies substituted with HERS, the 30RSC exhibited high levels of SDS and RS, along with desirable physical characteristics and organoleptic profiles. The visual appearance of 30RSC became more appealing, closely resembling that of WSC, which enhanced its consumer appeal, achieving an average score of 7 on a 9‐point scale. Furthermore, the physical, chemical, and mechanical properties of 30RSC were comparable to those of WSC, demonstrating the stability of the production process and the quality of the final product. Regarding starch digestibility, 30RSC resulted in lower blood glucose release after ingestion, classifying it as a low‐GI gluten‐free food with reduced calorie content. Further human trials should be conducted on the rice‐based cookies substituted with 30% HERS to assess their potential suitability for overweight and diabetic individuals.

**Nomenclature**
15RSC: cookies prepared from rice flour substituted with 15% HERS using stevia
20RSC: cookies prepared from rice flour substituted with 20% HERS using stevia
25RSC: cookies prepared from rice flour substituted with 25% HERS using stevia
30RSC: cookies prepared from rice flour substituted with 30% HERS using stevia
35RSC: cookies prepared from rice flour substituted with 35% HERS using stevia
40RSC: cookies prepared from rice flour substituted with 40% HERS using stevia
45RSC: cookies prepared from rice flour substituted with 45% HERS using stevia
GFC: gluten‐free cookies
GI: glycemic index
HERS: highly enzyme‐resistant mung bean starch
RC: cookies prepared from 100% rice flour using stevia
RDS: rapidly digestible starch
RS: resistant starch
SDS: slowly digestible starch
WC: cookies prepared from 100% wheat flour using stevia
WSC: cookies prepared from 100% wheat flour using sugar

## Ethics Statement

The white mice (*Mus musculus domesticus*) of the Swiss line were supplied by the Pasteur Institute in Ho Chi Minh City in Vietnam. All steps in the assay to quantify the blood glucose response in mice were approved by the Institutional Animal Care and Use Committee of the International University, Vietnam National University in Ho Chi Minh City, Vietnam.

## Consent

Written consent was obtained after participants were informed of the research objectives.

## Conflicts of Interest

The authors declare no conflicts of interest.

## Author Contributions

Nguyen Thi Mai Huong and Nguyen Ngoc Thanh Tien: writing—original draft, writing—review and editing, conceptualization, methodology, data curation, and validation; Phan Ngoc Hoa and Nguyen Thi Lan Phi: conceptualization, methodology, resources, and supervision; Pham Van Hung: investigation, conceptualization, methodology, validation, resources, writing—original draft, writing—review and editing, project administration, and supervision.

## Funding

The study was supported by Viet Nam National University Ho Chi Minh City, (A2024‐28‐02).

## Data Availability

The data that support the findings of this study are available from the corresponding author upon reasonable request.
